# Molecular epidemiology of carbapenemase-producing gram-negative bacilli in diabetic foot infections: association with glycemic control

**DOI:** 10.3389/fcimb.2026.1847743

**Published:** 2026-10-05

**Authors:** Gamal Othman, Abdelnaser A. Badawy, Rasha Hamed Al-Serwi, Maysaa El Sayed Zaki, Noha Tharwat Abou El-khier, Nashwa M. Alkassaby, Mohamed Saleh Ismail

**Affiliations:** 1 Department of Basic Medical Sciences, College of Medicine, AlMaarefa University, Riyadh, Saudi Arabia; 2 Department of Biochemistry, Faculty of Medicine, Northern Border University, Arar, Saudi Arabia; 3 Department of Basic Dental Sciences, College of Dentistry, Princess Nourah bint Abdulrahman University, Riyadh, Saudi Arabia; 4 Clinical Pathology-Mansoura Faculty of Medicine, Mansoura University, Mansoura, Egypt; 5 Medical Microbiology and Immunology- Mansoura Faculty of Medicine, Mansoura University, Mansoura, Egypt

**Keywords:** antimicrobial stewardship, blaKPC, BlaNDM, carbapenem resistance, diabetic foot ulcer, gram-negative bacilli, HbA1c, *Klebsiella pneumoniae*

## Abstract

**Background:**

Carbapenem-resistant Gram-negative bacilli are an increasing threat in diabetic foot ulcer (DFU) infections, limiting therapeutic options and worsening outcomes. However, data linking specific carbapenemase genes to clinical and metabolic factors remain limited.

**Objectives:**

To isolate Gram-negative bacilli from deep tissue biopsies of DFUs, determine carbapenem susceptibility by minimum inhibitory concentration (MIC) testing, detect major carbapenemase genes by multiplex qPCR, and evaluate their association with glycemic control and Wagner grade.

**Methods:**

This cross-sectional study included 150 patients with infected DFUs. Isolates recovered from tissue and other specimens were identified by standard microbiological methods, and carbapenem susceptibility was assessed by MIC testing. Multiplex qPCR was used to detect *bla*KPC, *bla*NDM, *bla*OXA-48, *bla*VIM, and *bla*IMP, and associations between resistance determinants and clinical variables were analyzed statistically.

**Results:**

The mean patient age was 56.4 ± 9.8 years, with a male predominance (61.3%), and high-grade ulcers (Wagner III–V) were present in 54.7% of patients. *Klebsiella pneumoniae* predominated (60.0%), followed by *Pseudomonas aeruginosa* and *Escherichia coli* (each 14.7%). Resistance to meropenem and imipenem was high (77.3% and 75.3%, respectively), and carbapenemase production was detected in 84.0% of isolates. *bla*KPC (28.0%) and *bla*NDM (24.0%) were the most prevalent genes and were significantly associated with phenotypic carbapenemase production (P = 0.002 and P = 0.006, respectively). Carbapenemase producers showed significantly higher fasting glucose and HbA1c levels than non-producers (P < 0.001), with no significant association observed for Wagner grade (P = 0.570).

**Conclusions:**

DFUs showed a high burden of carbapenemase-producing Gram-negative bacilli, driven mainly by *bla*KPC and *bla*NDM. Poor glycemic control was strongly associated with these resistant infections, underscoring the need for integrated metabolic management, molecular surveillance, and antimicrobial stewardship. These findings position glycemic control as a modifiable driver of antimicrobial resistance in DFUs.

## Introduction

Diabetic foot ulcers (DFUs) represent one of the most debilitating chronic complications of diabetes mellitus, affecting approximately 19% to 34% of diabetic patients during their lifetime (Everett and Mathioudakis, 2018). These lesions often serve as a portal for complex, polymicrobial infections that can progress rapidly to osteomyelitis, gangrene, and lower-extremity amputation (Schaper et al., 2024). In 2025, systematic reviews highlighted an epidemiological shift in diabetic foot infections (DFIs) toward Gram-negative bacilli (GNB), which now predominate in many regions across Asia and Africa, accounting for up to 71% of clinical isolates (Arjan et al., 2025; Zhang et al., 2025).

The management of GNB in diabetic wounds is severely compromised by the global escalation of antimicrobial resistance (AMR). Among these, carbapenem-resistant Gram-negative bacilli (CR-GNB)—including carbapenem-resistant Enterobacterales (CRE) and *Pseudomonas aeruginosa* (*P. aeruginosa*)—have been designated by the World Health Organization (WHO) as “critical priority” pathogens (World Health Organization, 2024). Recent multi-center studies in 2025 reported that over 51% of GNB isolated from DFUs were confirmed carbapenemase producers, with *Klebsiella pneumoniae* (*K. pneumoniae*) and *Escherichia coli* (*E. coli*) emerging as the most frequent carriers of resistance genes such as *bla*KPC and *bla*NDM (Saleem et al., 2025).

The clinical impact of carbapenem resistance in the diabetic population is profound. Patients harboring CR-GNB are significantly more likely to experience prolonged hospitalizations (averaging 27.9 days) and to exhibit a significantly higher risk of major amputation compared with those with carbapenem-susceptible infections (Braun and Silva, 2023; Ngono et al., 2025). Furthermore, the hyperglycemic environment characteristic of diabetes has been found to upregulate the virulence of these pathogens, specifically enhancing biofilm formation and capsule thickness in *K. pneumoniae*, which further shields the bacteria from both host immunity and last-resort antibiotics (P SR et al., 2024).

Despite the introduction of novel β-lactam/β-lactamase inhibitor combinations, such as ceftazidime-avibactam and the siderophore cephalosporin cefiderocol, therapeutic options for CR-GNB in DFIs remain limited and costly (Tamma et al., 2024a). Given the rapid dissemination of these resistant clones within healthcare settings, there is an urgent need for localized microbiological surveillance and molecular characterization of resistance determinants. This study aims to investigate the prevalence, genetic profile, and clinical outcomes of carbapenem-resistant Gram-negative bacilli in patients with diabetic foot infections to inform more effective empirical and targeted treatment protocols.

While the prevalence of carbapenemase-producing GNB in diabetic foot infections has been documented, a critical knowledge gap remains regarding the molecular synergy between coexisting pathogens and the impact of host glycemic volatility on gene expression. Furthermore, the clinical significance of genotypic heteroresistance, which often evades standard diagnostic protocols, remains underexplored in the context of lower-extremity limb salvage.

To our knowledge, this is the first study to integrate carbapenemase genotyping with glycemic status among DFU patients in Egypt.

The objectives of the present study were to:

- Isolate Gram-negative bacilli from deep tissue biopsies and determine their susceptibility patterns to carbapenems using MIC determination and multiplex qPCR for the *bla*KPC, *bla*NDM, *bla*OXA-48, *bla*VIM, and *bla*IMP genes.
- Analyze the association between the presence of specific carbapenemase genes and clinical parameters, including glycemic control (HbA1c levels), Wagner ulcer grade, and history of prior antibiotic exposure.

## Materials and methods

### Study design and participants

This cross-sectional study was conducted at Mansoura University Hospital, Egypt, between March and July 2026 and enrolled adult patients presenting with infected diabetic foot ulcers (DFUs).

Eligibility required patients aged ≥18 years with a confirmed diagnosis of type 1 or type 2 diabetes mellitus and a diabetic foot infection classified as Wagner grade ≥2 or IWGDF/IDSA grade 3–4 (moderate to severe). Patients were excluded if they had superficial ulcers (Wagner grade 1), isolated cellulitis without deep tissue involvement, or had received systemic antibiotic therapy within the preceding 7 days (European Center for Disease Prevention and Control, 2024).

The study protocol was approved by the Institutional Research Board/Ethics Committee of the Faculty of Medicine, Mansoura University, Egypt (R.26.03.3606), and the study was conducted in accordance with the ethical principles of the Declaration of Helsinki. All participants provided written informed consent before enrollment.

### Sample size calculation

Sample size was calculated to estimate the prevalence of carbapenemase-producing Gram-negative bacilli among patients with diabetic foot infections, using the single-proportion formula:


$$\text{n}={\text{Z}}^{2}\times \text{p}\times (1-\text{p})/{\text{d}}^{2}$$

where n represents the required sample size, Z the standard normal deviate at the 95% confidence level (1.96), p the expected prevalence, and d the margin of error. In the absence of reliable local estimates, prevalence was assumed at 50%, and precision was set at 8%, yielding a minimum requirement of 150 patients. All 150 eligible patients were subsequently enrolled consecutively over the study period.

### Clinical and laboratory assessment

For each participant, clinical data were recorded, including age, sex, diabetes duration, prior antibiotic exposure, comorbidities, Wagner ulcer grade, and clinical outcome.

After overnight fasting, venous blood was drawn to measure fasting blood glucose and glycated hemoglobin (HbA1c). Fasting glucose was determined by the glucose oxidase–peroxidase method on a fully automated clinical chemistry analyzer (Dialab GmbH, Austria), while HbA1c was quantified using a commercial ELISA kit (Bioassay Technology Laboratory, Shanghai, China) per the manufacturer’s instructions; internal quality control was maintained throughout.

### Specimen collection and microbiological identification

Wounds were cleansed with sterile normal saline and debrided as needed prior to aseptic specimen collection, thereby minimizing the risk of contamination. Specimens were obtained using an aseptic technique after cleansing the wound surface with sterile normal saline to remove loose debris and superficial contaminants. This was followed by appropriate surgical debridement of devitalized, necrotic, and contaminated tissue. Deep-tissue biopsy, the preferred sampling method in current diabetic foot infection guidelines, was used throughout. Specimens were promptly transported to the Microbiology Laboratory, where they were inoculated onto blood, MacConkey, and chocolate agar and incubated aerobically at 37 °C for 24–48 hours.

Isolates were identified by colony morphology, Gram staining, and standard biochemical testing, supplemented by automated identification systems where available. Only Gram-negative bacilli recovered from clinically significant infections were retained for further analysis.

### Antimicrobial susceptibility testing

Minimum inhibitory concentrations (MICs) were determined by broth microdilution for imipenem, meropenem, colistin, ceftazidime, cefepime, piperacillin–tazobactam, ciprofloxacin, levofloxacin, and amikacin. Results were interpreted according to CLSI M100 (35th edition, 2025) breakpoints, with colistin interpreted per CLSI/EUCAST recommendations; full breakpoint criteria appear in **Supplementary Table S1**.

### Phenotypic detection of carbapenemase production

Carbapenemase production was first screened using the double-disk synergy test (DDST): a meropenem (10 µg) disk was placed on Mueller–Hinton agar inoculated with the test organism, alongside inhibitor disks containing phenylboronic acid and EDTA at standardized distances. Phenylboronic acid identified class A carbapenemases (e.g., KPC), while EDTA identified metallo-β-lactamases (e.g., NDM, VIM, and IMP); enhancement of the inhibition zone toward either inhibitor disk was scored as positive.

Carbapenem-resistant isolates then underwent confirmatory testing by the modified carbapenem inactivation method (mCIM) and EDTA-modified carbapenem inactivation method (eCIM), per CLSI 2025 recommendations, allowing both confirmation of carbapenemase production and differentiation of metallo-β-lactamase producers (Clinical and Laboratory Standards Institute, 2025).

### Molecular detection of carbapenemase genes

Multiplex real-time PCR (qPCR) was used to detect five major carbapenemase genes—blaKPC, blaNDM, blaOXA-48, blaVIM, and blaIMP—in carbapenem-resistant isolates. Genomic DNA was extracted from pure overnight cultures by the boiling method, and amplification was carried out with previously validated gene-specific primers and hydrolysis probes.

Each 20 μL reaction contained multiplex master mix, primer/probe mixture, template DNA, and nuclease-free water. Cycling conditions comprised initial denaturation at 95 °C for 3 minutes, followed by 40 cycles of 95 °C for 15 seconds and 60 °C for 60 seconds (annealing/extension) (Monteiro et al., 2012). Positive and negative controls accompanied every run, and samples were scored positive when they produced specific amplification curves with cycle threshold values within the validated range. Primer and probe sequences are listed in **Table 1** (Khan et al., 2025; Nadkarni et al., 2002; Pickwell et al., 2013a; Poirel et al., 2011; Prompers et al., 2007; Sisay et al., 2025b).

**Table 1:** Multiplex qPCR primer and probe master list.

Target gene	Primer/probe	Sequence (5’–3’)	Reporter dye	Quencher
*blaKPC​*	Forward Primer	ATGTCACTGTATCGCCGTCT	–	–
	Reverse Primer	TTTTCAGAGCCTTACTGCCC	–	–
	Probe	CCGACCGGCAGCAGTTTGTT	FAM	BHQ-1
*blaNDM​*	Forward Primer	GGTTTGGCGATCTGGTTTTC	–	–
	Reverse Primer	CGGAATGGCTCATCACGATC	–	–
	Probe	CCGGTGGCATTGATAAGCTC	HEX/VIC	BHQ-1
*blaOXA−48​*	Forward Primer	GCGTGGTTAAGGATGAACAC	–	–
	Reverse Primer	CATCAAGTTCAACCCAACCG	–	–
	Probe	AGCGCTCCCGTGGTTTCA	ROX/Texas Red	BHQ-2
*blaVIM​*	Forward Primer	GTTTGGTCGCATATCGCAAC	–	–
	Reverse Primer	TCGATGGTGTTTGGTCGCAT	–	–
	Probe	CCTCGATGGTGTTTGGTCGC	Cy5	BHQ-2
*blaIMP​*	Forward Primer	GGAATAGAGTGGCTTAAYTCTC	–	–
	Reverse Primer	CCAAAYTTAGTTGAYTTAAYTT	–	–
	Probe	ATCCATCTGCATCCGTT	Cy5.5/Q705	BHQ-3
Internal Control	Forward/Reverse	(e.g., 16S rRNA or PhHV)	–	–
	Probe	Custom Reference Sequence	TAMRA/NED	BHQ-2

### Statistical analysis

Data were analyzed using SPSS software version 28.0 (IBM Corp., Armonk, NY, USA). Continuousvariables were expressed as mean ± standard deviation (SD) or median (interquartile range), asappropriate, while categorical variables were expressed as frequencies and percentages. Categorical variables were compared using the Chi-square test or Fisher’s exact test, as appropriate, and continuous variables were compared using the Mann–Whitney U test for non-normally distributed data. A two-tailed P value < 0.05 was considered statistically significant (Khan et al., 2025; Nadkarni et al., 2002; Pickwell et al., 2013a; Poirel et al., 2011; Prompers et al., 2007; Sisay et al., 2025).

## Results

### Patient characteristics and clinical profile

The study enrolled 150 patients with infected diabetic foot ulcers, with a mean age of 56.4 ± 9.8 years and a male predominance (61.3% vs. 38.7% female).

Wagner grade II (25.3%) and grade III (24.7%) were the most common severity categories; when grouped, high-grade ulcers (Wagner III–V) accounted for the majority (54.7%) compared with low-grade ulcers (grades I–II, 45.3%).

Tissue specimens accounted for the largest share of samples collected (40.7%), followed by swabs (30.7%) and pus (20.0%), with bone biopsy the least common (8.6%) (**Table 2**).

**Table 2:** Demographic, clinical, and laboratory findings in patients.

Variable	Value
Age (years)	56.4 ± 9.8
Sex	
Male	92 (61.3%)
Female	58 (38.7%)
Wagner ulcer grade	
Grade I	30 (20.0%)
Grade II	38 (25.3%)
Grade III	37 (24.7%)
Grade IV	30 (20.0%)
Grade V	15 (10.0%)
Ulcer severity (grouped)	
Low grade (I–II)	68 (45.3%)
High grade (III–V)	82 (54.7%)
Sample type	
Tissue	61 (40.7%)
Swab	46 (30.7%)
Pus	30 (20.0%)
Bone biopsy	13 (8.6%)
Fasting blood glucose (mg/dL)	
Overall	158.6 ± 41.2
Low-grade ulcers (I–II)	139.8 ± 32.4
High-grade ulcers (III–V)	174.9 ± 38.1
HbA1c (%)	
Overall	8.1 ± 1.4
Low-grade ulcers (I–II)	7.4 ± 1.0
High-grade ulcers (III–V)	8.7 ± 1.3

Mean fasting blood glucose was 158.6 ± 41.2 mg/dL, and mean HbA1c was 8.1 ± 1.4%; both were higher among patients with high-grade ulcers than those with low-grade ulcers (**Table 2**). Stratifying by specimen type showed no significant association with phenotypic carbapenemase production (χ² = 0.10, P = 1.00), suggesting that specimen type did not influence microbiological findings (**Supplementary Table S2**).

### Antimicrobial resistance profile

Antimicrobial resistance was widespread among the isolated Gram-negative bacilli (**Figure 1**). Resistance to carbapenems was especially pronounced, with 77.3% of isolates resistant to meropenem and 75.3% to imipenem. Resistance was also high for piperacillin–tazobactam (62.0%), ceftazidime (58.0%), and cefepime (55.3%), and moderate for the fluoroquinolones and aminoglycosides tested—ciprofloxacin (56.0%), amikacin (54.0%), and levofloxacin (50.0%). Colistin resistance, while the lowest recorded (51.3%), remained substantial.

**Figure 1:**
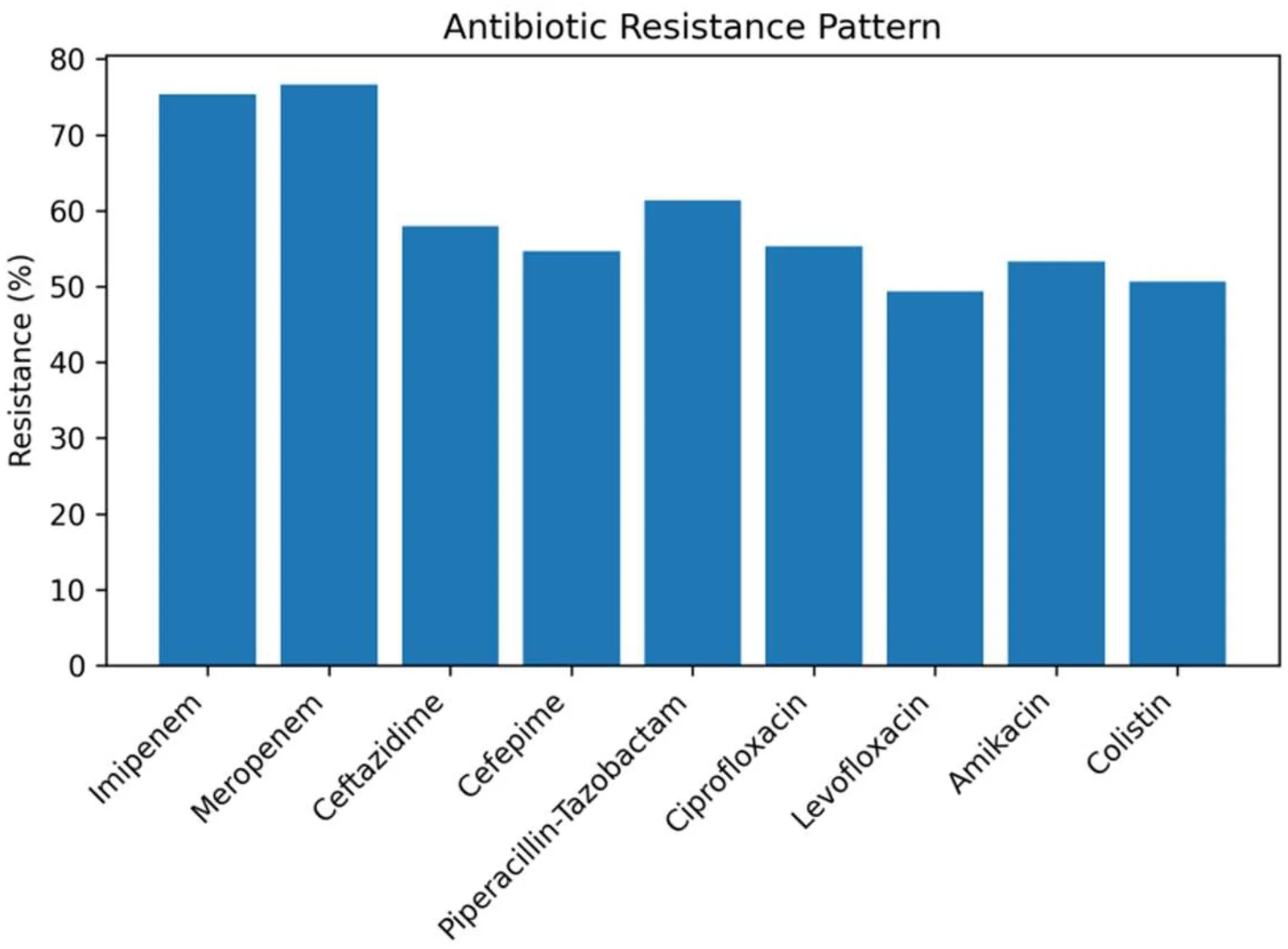
Antibiotic resistance among the isolated Gram-negative bacilli.

### Distribution of bacterial isolates

GNB predominated overall, led by *K. pneumoniae* (60.0%). *P. aeruginosa* and *E. coli* each accounted for 14.7%, and *Serratia marcescens* for 10.6% (**Table 3**).

**Table 3:** Distribution of isolated bacterial species.

Category/isolate characteristic	Frequency (n)	Percentage (%)
Phenotypic Carbapenemase Status		
Carbapenemase Producer	126	84.0%
Non-Carbapenemase Producer	24	16.0%
Isolated Bacterial Species		
*Klebsiella pneumoniae*	90	60.0%
*Pseudomonas aeruginosa*	22	14.7%
*Escherichia coli*	22	14.7%
*Serratia marcescens*	16	10.6%

### Phenotypic carbapenemase production

Carbapenemase production was common: of the 150 isolates, 126 (84.0%) tested positive phenotypically, while the remaining 24 (16.0%) were non-producers (**Table 3**).

### Distribution of carbapenemase genes

Carbapenemase genes were unevenly distributed among the isolates (**Table 4**). *bla*KPC was the most frequently detected gene (42 isolates, 28.0%), followed by *bla*NDM (36 isolates, 24.0%); *bla*VIM and *bla*IMP were each identified in 14 isolates (9.3%), and *bla*OXA-48 was the least common, found in only 6 isolates (4.0%).

**Table 4:** Frequency of detected carbapenemase genes among the studied isolates (n = 150).

Carbapenemase gene	Number of isolates	Percentage
*blaKPC*	42	28.0%
*blaNDM*	36	24.0%
*blaOXA-48*	6	4.0%
*blaVIM*	14	9.3%
*blaIMP*	14	9.3%

Most carbapenemase-producing isolates (92, 73.0%) carried a single gene, while 34 (27.0%) harbored multiple genes—most often *bla*KPC + *bla*NDM (n = 16) and *bla*NDM + *bla*VIM (n = 8). Isolates with multiple carbapenemase genes showed higher MIC values for both imipenem and meropenem than those with a single gene.

### Association between phenotypic carbapenemase production and carbapenemase genes

Phenotypic carbapenemase production correlated significantly with the presence of *bla*KPC (χ² = 9.52, P = 0.002) and *bla*NDM (χ² = 7.52, P = 0.006), but not with *bla*OXA-48, *bla*VIM, or *bla*IMP (P > 0.05 for all) (**Table 5**).

**Table 5:** Association between phenotypic carbapenemase production and carbapenemase genes.

Carbapenemase gene	χ² value	p value	Significance
*blaKPC*	9.52	0.002	Significant
*blaNDM*	7.52	0.006	Significant
*blaOXA-48*	0.27	0.601	Not significant
*blaVIM*	1.77	0.183	Not significant
*blaIMP*	1.77	0.183	Not significant

### Association between carbapenemase production and glycemic control

Glycemic control was significantly poorer among patients with carbapenemase-producing isolates than among those without (**Table 6**). Fasting blood glucose was higher in the carbapenemase-producing group (176.1 ± 37.3 mg/dL) than in the non-producing group (123.9 ± 18.7 mg/dL, P < 0.001), as was HbA1c (8.4 ± 1.20% vs. 7.00 ± 0.9%, P < 0.001).

**Table 6:** Association between phenotypic carbapenemase production and clinical parameters.

Parameter/variable	Carbapenemase producers (n=126)	Non-producers (n=24)	Statistical test	p-value
Glycemic & clinical parameters				
Fasting blood glucose (mg/dL), Mean ± SD	176.1 ± 37.3	123.9 ± 18.7	Mann–Whitney U	< 0.001
HbA1c (%), Mean ± SD	8.4 ± 1.20	7.00 ± 0.9	Mann–Whitney U	< 0.001
Duration of diabetes (years), Mean ± SD	12.4 ± 5.3	7.8 ± 3.6	Mann–Whitney U	< 0.001
Wagner classification – Low grade (≤2), n (%)	52 (41.3%)	12 (50.0%)	Chi-square	0.570
Wagner classification – High grade (>2), n (%)	74 (58.7%)	12 (50.0%)	Chi-square	0.570
Risk factors & comorbidities				
Prior antibiotic exposure, n (%)	95 (75.4%)	8 (33.3%)	Chi-square	< 0.001
Hypertension, n (%)	89 (70.6%)	10 (41.7%)	Chi-square	0.006
Chronic kidney disease, n (%)	35 (27.8%)	2 (8.3%)	Fisher’s exact	0.048
Peripheral vascular disease, n (%)	51 (40.5%)	3 (12.5%)	Chi-square	0.009
Ischemic heart disease, n (%)	27 (21.4%)	2 (8.3%)	Fisher’s exact	0.145
Clinical outcomes				
Amputation, n (%)	42 (33.3%)	2 (8.3%)	Chi-square	0.015
Hospitalization duration (days), Mean ± SD	14.6 ± 4.8	8.1 ± 2.9	Mann–Whitney U	< 0.001

Data are presented as numbers (%) or means ± standard deviation (SD).

Chi-square test was used for categorical variables with expected counts ≥5.

Fisher’s exact test was used for categorical variables with expected counts <5.

The Mann–Whitney U test was used to compare non-normally distributed continuous variables.

P < 0.05 was considered statistically significant.

Carbapenemase production was not significantly associated with Wagner-classified ulcer severity (P = 0.570), but it was significantly associated with prior antibiotic exposure (P < 0.001) and longer diabetes duration (P < 0.001). Among comorbidities, significant associations emerged for hypertension (P = 0.006), chronic kidney disease (P = 0.048), and peripheral vascular disease (P = 0.009), but not for ischemic heart disease (P = 0.15).

Clinically, carbapenemase-producing infections were associated with significantly higher amputation rates (P = 0.015) and longer hospital stays (P < 0.001) than non-producing infections.

### Association between multiple carbapenemase genes and carbapenem MIC values

Isolates harboring multiple carbapenemase genes showed significantly higher imipenem and meropenem MIC values than those with a single gene. Median MICs rose from 16 µg/mL (IQR: 8–32) in single-gene isolates to 64 µg/mL (IQR: 32–128) in multi-gene isolates for both carbapenems (P < 0.001), indicating markedly greater resistance associated with co-existing carbapenemase genes (**Table 7**).

**Table 7:** Association between multiple carbapenemase genes and carbapenem MIC values.

Variable	Single carbapenemase (n=88)	Multiple carbapenemases (n=12)	Test	P value
Imipenem MIC (µg/mL), median (IQR)	16 (8–32)	64 (32–128)	Mann–Whitney U	<0.001
Meropenem MIC (µg/mL), median (IQR)	16 (8–32)	64 (32–128)	Mann–Whitney U	<0.001

MIC, minimum inhibitory concentration; IQR, interquartile range.

MIC values were generated using standard doubling dilutions (8, 16, 32, 64, and 128 µg/mL).

P < 0.05 was considered statistically significant.

### Longitudinal glycemic control and carbapenemase-producing infection

Longitudinal assessment of glycemic control showed consistently higher HbA1c levels among patients with carbapenemase-producing Gram-negative infections than among those with non-carbapenemase-producing infections (**Table 8**). The HbA1c value closest to ulcer presentation was significantly higher among carbapenemase producers than among non-producers (8.4 ± 0.8% vs. 7.05 ± 0.8%, respectively; Welch’s *t* = 7.61, *P* < 0.001). A similar difference was observed for mean HbA1c during the preceding 12 months, which was 8.4 ± 0.80% in the carbapenemase-producing group compared with 6.9 ± 0.7% in the non-producing group (Welch’s *t* = 9.03, *P* < 0.001). Maximum HbA1c during the preceding year was also significantly higher among carbapenemase producers (8.81 ± 0.82% vs. 7.3 ± 0.8%; Welch’s *t* = 7.91, *P* < 0.001).

**Table 8:** Longitudinal glycemic-control measures according to carbapenemase-production status.

Variable	Carbapenemase producers (n=126)	Non-producers (n=24)	Test statistic	P value
HbA1c closest pre-ulcer, %, mean ± SD	8.4 ± 0.8	7.05 ± 0.82	Welch t = 7.61	<0.001
Mean HbA1c during previous 12 months, %, mean ± SD	8.4 ± 0.80	6.96 ± 0.71	Welch t = 9.03	<0.001
Maximum HbA1c during previous 12 months, %, mean ± SD	8.8± 0.8	7.34 ± 0.8	Welch t = 7.91	<0.001
Proportion of HbA1c measurements ≥8%, mean ± SD	70.6 ± 37.8%	11.5 ± 20.%	Welch t = 10.91	<0.001
Patients with ≥50% of prior HbA1c measurements ≥8%, n (%)	98 (77.8%)	5 (20.8%)	χ² = 27.79	<0.001

Welch’s independent-samples t test was used for continuous variables; χ² test was used for the categorical comparison. HbA1c, glycated hemoglobin.

The proportion of HbA1c measurements ≥8% during the preceding 12 months was substantially greater among patients with carbapenemase-producing infections (70.6 ± 37.8%) than among those with non-producing isolates (11.5 ± 20.8%; Welch’s *t* = 10.91, *P* < 0.001). Furthermore, 98/126 (77.8%) patients in the carbapenemase-producing group had at least 50% of their preceding HbA1c measurements ≥8%, compared with 5/24 (20.8%) patients in the non-producing group (χ² = 27.79, *P* < 0.001). Collectively, these findings indicate a strong association between persistently poorer glycemic control during the year preceding presentation and carbapenemase-producing infection.

In univariable logistic regression analysis (**Table 9**), increasing longitudinal HbA1c measures were consistently associated with greater odds of carbapenemase-producing infection. Each 1% increase in the HbA1c measurement closest to ulcer presentation was associated with an OR of 8.14 (95% CI, 3.58–18.48; *P* < 0.001), whereas each 1% increase in mean HbA1c during the preceding 12 months was associated with an OR of 15.72 (95% CI, 5.46–45.29; *P* < 0.001). Similarly, each 1% increase in maximum HbA1c during the preceding year was associated with an OR of 10.65 (95% CI, 4.31–26.29; *P* < 0.001).

**Table 9:** Logistic regression for association of longitudinal HbA1c measures with carbapenemase-producing infection.

Predictor	Unit/contrast	Odds ratio	95% CI	P value
HbA1c closest pre-ulcer	Per 1% increase	8.14	3.5–18.4	<0.001
Mean HbA1c during previous 12 months	Per 1% increase	15.72	5.4–45.2	<0.001
Maximum HbA1c during previous 12 months	Per 1% increase	10.65	4.3–26.2	<0.001
Proportion of HbA1c measurements ≥8%	0 to 1 increase	93.29	14.4–603.5	<0.001
≥50% of prior HbA1c measurements ≥8%	Yes vs No	13.30	4.5–38.8	<0.001

Each continuous predictor was fitted in a separate univariable logistic-regression model with carbapenemase-production status as the outcome. The ≥50% variable is reported as an unadjusted odds ratio.

Patients in whom at least 50% of HbA1c measurements during the preceding year were ≥8% had 13.30-fold higher odds of carbapenemase-producing infection compared with those below this threshold (OR, 13.30; 95% CI, 4.56–38.81; *P* < 0.001), **Table 8**.

## Discussion

Recent molecular studies implicate carbapenemase-producing GNB—particularly *Klebsiella pneumoniae*, *Pseudomonas aeruginosa*, *Acinetobacter baumannii*, and *Escherichia coli*—with increasing frequency in diabetic foot ulcer (DFU) infections, with *bla*KPC and *bla*NDM predominating among resistance determinants and substantially complicating both therapy and clinical outcomes (Saleem et al., 2025).

The demographic profile of our cohort—mean age in the mid-fifties with marked male predominance—aligns with the well-established epidemiology of DFUs, which disproportionately affect older men owing to higher rates of peripheral neuropathy, peripheral vascular disease, and behavioral risk factors (Armstrong et al., 2017; Lipsky et al., 2020). The predominance of Wagner grades II–III suggests delayed clinical presentation, a pattern frequently reported in hospital-based DFU populations (Lipsky et al., 2020), while the substantial proportion of advanced ulcers reflects a high burden of severe disease at presentation—consistently linked to greater infection risk, delayed healing, and poorer outcomes (Armstrong et al., 2017).

The predominance of tissue specimens in this study accords with current recommendations favoring deep tissue sampling for microbiological evaluation of infected DFUs: compared with superficial swabs, tissue specimens more accurately represent causative pathogens, particularly in chronic or advanced wounds, and improve the reliability of antimicrobial susceptibility testing (Nelson et al., 2025).

Poor glycemic control was strongly associated with ulcer severity. Elevated fasting blood glucose and HbA1c levels among patients with high-grade ulcers corroborate prior evidence that chronic hyperglycemia drives ulcer progression through impaired immune function, reduced leukocyte activity, and microvascular compromise (Armstrong et al., 2017; Zhang et al., 2017), facilitating persistent infection and delayed repair and underscoring the central role of glycemic optimization in DFU prevention and management.

The high level of antimicrobial resistance observed among GNB in this study reflects the escalating global burden of multidrug resistance in DFIs. Comparable patterns have been documented in recent surveillance studies, where *E. coli* and *P. aeruginosa* frequently show resistance to multiple antibiotic classes (Das et al., 2025; Li et al., 2025a), including—consistent with our findings—diminished susceptibility to broad-spectrum β-lactams such as piperacillin–tazobactam, ceftazidime, and cefepime (Das et al., 2025). Rising resistance to fluoroquinolones and aminoglycosides has also been reported, likely driven by the high prevalence of extended-spectrum β-lactamases and other resistance determinants in DFU isolates (Coşkun et al., 2024; Das et al., 2025). Of particular concern, emerging colistin resistance—even among multidrug-resistant strains—further narrows therapeutic options and underscores the need for antimicrobial stewardship and routine susceptibility testing in DFU management (Coşkun et al., 2024; Das et al., 2025). Together, these findings highlight the necessity of tailoring empirical therapy to local resistance patterns and integrating stewardship with glycemic optimization.

Microbiological analysis demonstrated a clear predominance of Gram-negative bacilli, led by *K. pneumoniae*, followed by *P. aeruginosa*, *E. coli*, and *Serratia marcescens*—a distribution consistent with recent reports of a shift toward Gram-negative dominance in DFIs, particularly in chronic and advanced ulcers (Maity et al., 2024; Yan et al., 2025). Several 2024–2025 studies likewise identify *K. pneumoniae* as a principal DFU pathogen frequently associated with multidrug resistance and carbapenemase production (Das et al., 2025; Yan et al., 2025). The prominence of *P. aeruginosa* and *E. coli* in our cohort mirrors contemporary hospital-based surveillance data (Maity et al., 2024; Reddy et al., 2024); both organisms pose particular clinical challenges through biofilm formation, persistence in chronic wounds, and accumulation of multiple resistance mechanisms, contributing to therapeutic failure and delayed healing (Reddy et al., 2024). Although less frequent, the recovery of *S. marcescens* is likewise consistent with recent reports describing its opportunistic role in severe or previously treated DFUs (Sisay et al., 2024; Das et al., 2025). Collectively, these findings reinforce the growing dominance of Gram-negative pathogens in DFIs and the importance of ongoing microbiological surveillance (Maity et al., 2024; Reddy et al., 2024; Sisay et al., 2024; Yan et al., 2025).

Phenotypic screening revealed a notably high prevalence of carbapenemase production (84.0%)—higher than most recent reports. A 2025 multicenter study, for example, found carbapenemase production in approximately 51.7% of DFU isolates (Pattolath et al., 2024), while other series report CRE rates of roughly 30–40% among Gram-negative foot isolates (Li et al., 2025a; Saleem et al., 2025), and meta-analyses similarly describe heterogeneous but generally lower pooled prevalence estimates (Pattolath et al., 2024). Several factors may explain this discrepancy: recruitment from a tertiary referral center and the inclusion of previously treated or chronically ill patients likely enriched our cohort for multidrug-resistant organisms (Liang et al., 2024a; Saleem et al., 2025); combining sensitive phenotypic screening with targeted PCR improves detection relative to routine susceptibility testing alone (Reddy et al., 2024); local epidemiology, including possible clonal dissemination of carbapenemase-producing *K. pneumoniae*, may have contributed to a transient institutional surge (World Health Organization, 2024); and prior exposure to broad-spectrum β-lactams or carbapenems, together with suboptimal infection control, remain established drivers of carbapenemase spread (Das et al., 2025; Li et al., 2025a).

The predominance of *bla*KPC and *bla*NDM among our isolates mirrors global resistance trends and underscores their central role in carbapenem resistance; their presence is strongly linked to multidrug resistance, restricted therapeutic options, prolonged hospitalization, and increased risk of adverse outcomes, including treatment failure and limb loss. These findings support routine phenotypic screening for carbapenemase activity in DFU isolates, complemented by rapid molecular confirmation where feasible, alongside strengthened antimicrobial stewardship and infection-prevention measures (Pattolath et al., 2024; Sisay et al., 2024).

The presence of multiple carbapenemase genes in a substantial proportion of isolates highlights the growing complexity of resistance mechanisms in diabetic foot infections. Co-harboring of these genes was associated with markedly higher carbapenem MIC values, indicating enhanced resistance and reduced susceptibility to last-line agents—consistent with recent reports linking multi-gene carriage to extreme drug resistance and poorer clinical outcomes.

We also found a significant association between carbapenemase production and poor glycemic control, which may be explained by several interconnected biological mechanisms. Chronic hyperglycemia impairs neutrophil chemotaxis, phagocytosis, and intracellular killing, weakening early bacterial clearance (Casqueiro and Alves, 2012; Berbudi et al., 2020), while promoting oxidative stress, endothelial dysfunction, and dysregulated inflammatory signaling that further compromise host defense and worsen infection outcomes (Singh and Singh, 2022). This combination of low-grade inflammation and impaired immune function creates conditions favorable to bacterial persistence and recurrent infection. Diabetes-associated microvascular dysfunction compounds these effects by reducing tissue perfusion and oxygen delivery, thereby limiting the reach of immune cells, nutrients, and antimicrobial agents to infected tissue, impairing healing, and promoting the tissue hypoxia that favors bacterial survival and biofilm formation (Armstrong et al., 2017; Zhang et al., 2017).

More broadly, dysglycemia drives vascular dysfunction through endothelial injury, chronic inflammation, oxidative stress, and impaired nitric oxide bioavailability, promoting vascular smooth muscle proliferation, arterial stiffness, and accelerated atherosclerotic change—all of which worsen peripheral artery disease and reduce tissue perfusion (Weber et al., 2001). In diabetic foot infections specifically, this impaired circulation limits oxygen delivery, delays healing, and restricts antibiotic penetration into infected tissue. Similar vascular compromise may extend to non-atherosclerotic conditions such as Moyamoya disease, where endothelial dysfunction, immune dysregulation, and abnormal vascular remodeling likewise drive disease progression (Weber et al., 2001; Demartini et al., 2022); emerging evidence suggests diabetes-related metabolic disturbance may further impair collateral vessel formation and promote ischemic injury in such complex cerebrovascular disorders. Taken together, these observations reinforce that strict glycemic control is essential not only for infection control but also for preserving systemic vascular function (Weber et al., 2001; Demartini et al., 2022).

Hyperglycemia may also directly enhance bacterial virulence: high-glucose environments have been shown to promote biofilm formation, capsule production, and bacterial adhesion, particularly in *K. pneumoniae* and *P. aeruginosa* (Martins et al., 2013; Saleem et al., 2025). Biofilms act as protective barriers against host immunity and antimicrobial agents, facilitating horizontal gene transfer and the persistence of multidrug-resistant organisms.

Collectively, these mechanisms offer a coherent biological explanation for the heightened susceptibility of poorly controlled diabetic patients to carbapenemase-producing infections and underscore the importance of strict glycemic control within integrated infection management. As illustrated in **Figure 2**, chronic hyperglycemia may act as a central driver linking impaired host immunity, biofilm formation, and horizontal gene transfer, thereby facilitating the dissemination of carbapenemase genes (*bla*KPC, *bla*NDM, *bla*OXA-48, *bla*VIM, *bla*IMP) and amplifying antimicrobial resistance. Integrated strategies encompassing glycemic control, antimicrobial stewardship, and molecular surveillance are essential to mitigate this process.

**Figure 2:**
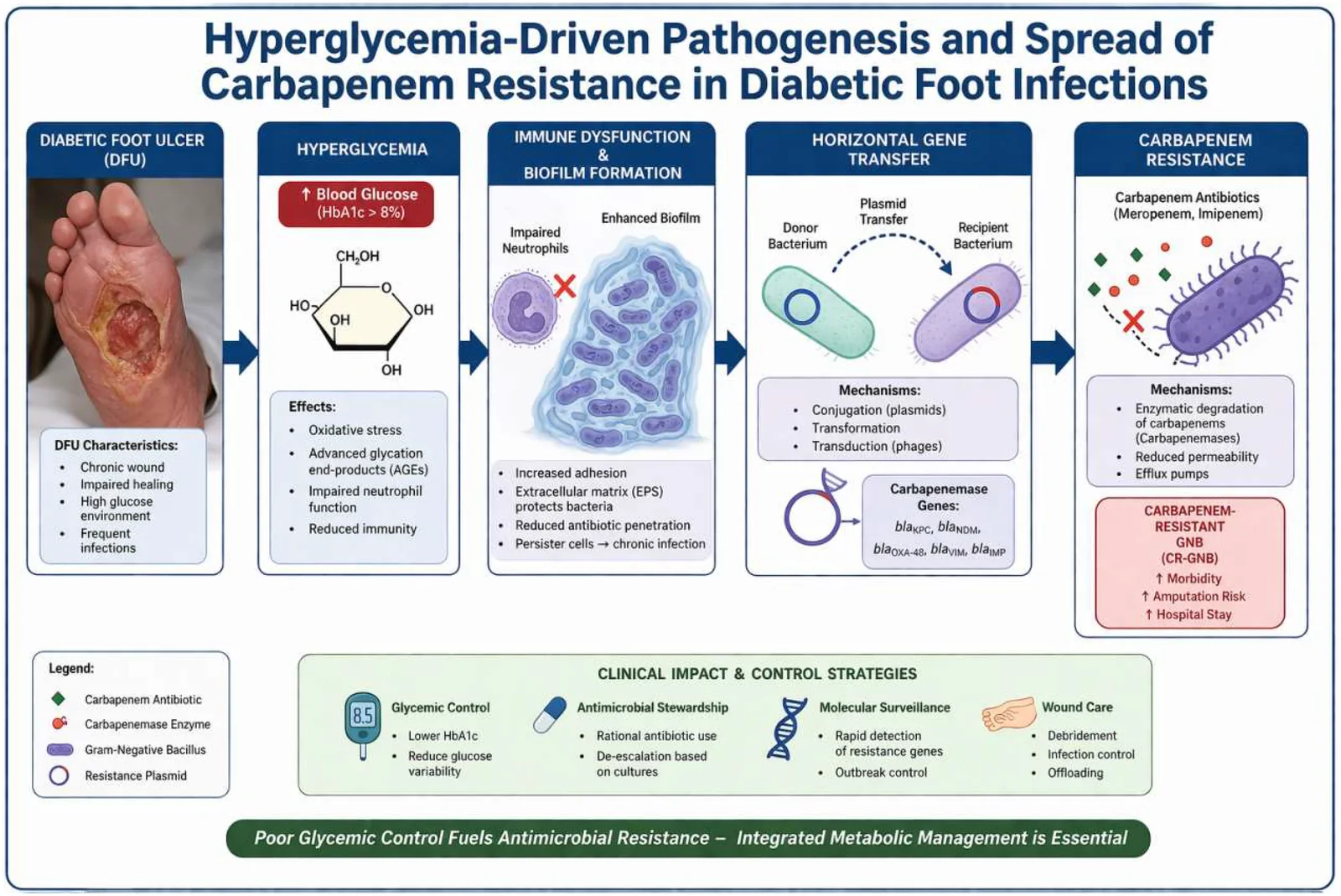
Proposed mechanistic pathway linking hyperglycemia to carbapenem resistance in diabetic foot infections.

In contrast, carbapenemase production was not significantly associated with Wagner grade (P = 0.570), suggesting that antimicrobial resistance is not directly determined by ulcer depth or clinical stage. The Wagner system primarily reflects anatomical severity rather than microbiological characteristics (Tiwari et al., 2012; Zhang et al., 2024), and recent studies similarly indicate that resistance patterns are more strongly influenced by prior antibiotic exposure, healthcare contact, and local epidemiology than by ulcer grade alone (Martins et al., 2013; Saleem et al., 2025).

Carbapenemase-producing Gram-negative bacilli in the present study were significantly associated with prior antibiotic exposure, longer duration of diabetes, hypertension, chronic kidney disease, peripheral vascular disease, higher amputation rates, and prolonged hospitalization—suggesting that resistance in diabetic foot infections is shaped not only by microbiological characteristics but also by host-related clinical factors.

Prior antibiotic exposure showed a particularly strong association with carbapenemase-producing isolates, consistent with previous reports that exposure to broad-spectrum antibiotics increases selective pressure and favors the emergence and persistence of multidrug-resistant organisms in chronic diabetic foot infections. Repeated antibiotic exposure may facilitate colonization by resistant Gram-negative pathogens, particularly carbapenemase producers, narrowing therapeutic options and increasing treatment failure; recent systematic reviews similarly identify prior antibiotic use and repeated hospitalization as major predictors of multidrug-resistant diabetic foot infections (Pickwell et al., 2013b; Hamzaoui et al., 2018a).

Longer diabetes duration was likewise associated with carbapenemase production. Chronic diabetes brings progressive neuropathy, microvascular dysfunction, impaired wound healing, and immune dysregulation, predisposing patients to recurrent, severe infection; patients with longstanding diabetes often require repeated hospital admissions and multiple antibiotic courses, raising their risk of acquiring resistant pathogens. Comparable observations appear in cohort studies linking disease chronicity to severe infection and antimicrobial resistance (Solgi et al., 2021a; WHO, 2024).

Among comorbidities, hypertension, chronic kidney disease, and peripheral vascular disease were each significantly associated with carbapenemase-producing infections. These conditions impair tissue perfusion, delay wound healing, and weaken immune responses, favoring chronic infection and the persistence of resistant organisms. Peripheral vascular disease is especially relevant, since ischemia limits delivery of both immune cells and antimicrobial agents to infected tissue, creating conditions conducive to bacterial persistence and biofilm formation; chronic kidney disease can further complicate therapy by altering drug pharmacokinetics and impairing host defense. Similar associations have been described in recent studies of risk factors for resistant diabetic foot infections (Bassetti et al., 2019; Solgi et al., 2021a; CDC, 2024; WHO, 2024).

Clinical outcomes underscored the burden of these infections: patients with carbapenemase-producing isolates had significantly higher amputation rates and longer hospital stays, consistent with reports linking multidrug-resistant GNB infection to worse outcomes and greater risk of major lower-limb amputation (Hamzaoui et al., 2018a; WHO, 2024). Ischemic heart disease, by contrast, showed no significant association with carbapenemase production—possibly reflecting the relatively small number of affected patients, or a more indirect contribution to resistance compared with vascular insufficiency, renal impairment, and prior antimicrobial exposure.

Taken together, these findings suggest that carbapenemase-producing infections in diabetic foot ulcers arise from a complex interaction between antimicrobial pressure, chronic metabolic dysregulation, vascular impairment, and host comorbidity. Early identification of high-risk patients based on these factors may support more targeted management, while integrated approaches combining antimicrobial stewardship, strict glycemic control, comorbidity optimization, and early, aggressive wound care remain essential to improving outcomes (Pickwell et al., 2013b; Hamzaoui et al., 2018a; Bassetti et al., 2019; Solgi et al., 2021a; CDC, 2024; WHO, 2024).

Isolates harboring multiple carbapenemase genes exhibited significantly higher imipenem and meropenem MIC values than those carrying a single gene—roughly a four-fold increase—suggesting that co-harboring multiple determinants may confer additive or synergistic resistance and markedly reduce susceptibility to last-line agents. The coexistence of multiple carbapenemase genes within a single isolate is an emerging clinical concern given its association with extreme drug resistance and severely limited therapeutic options; recent molecular studies show that Gram-negative bacilli carrying combinations of *bla*KPC, *bla*NDM, *bla*OXA-48, *bla*VIM, and *bla*IMP frequently exhibit greater resistance than single-gene isolates (Bassetti et al., 2019; CDC, 2024), with these determinants commonly plasmid-mediated and thus prone to horizontal transfer and rapid dissemination within healthcare settings. Similar findings—elevated MICs and extensive resistance phenotypes associated with co-expression of multiple carbapenemases—have been reported elsewhere (Liang et al., 2024b; Saleem et al., 2025; Sisay et al., 2025b), likely reflecting enhanced carbapenem hydrolysis when serine carbapenemases and metallo-β-lactamases are present together.

Clinically, isolates harboring multiple carbapenemase genes pose a serious therapeutic challenge in diabetic foot infections, where impaired tissue perfusion, chronic inflammation, and biofilm formation already limit antimicrobial penetration; such highly resistant pathogens carry an increased risk of treatment failure, prolonged hospitalization, and poor limb salvage (Tamma et al., 2024b; Li et al., 2025b; Saleem et al., 2025). These findings point to the growing complexity of carbapenem resistance mechanisms in this setting and underscore the need for continuous molecular surveillance, early detection of co-harboring isolates, and targeted stewardship to limit further spread.

Notably, a subset of carbapenem-resistant isolates in this study carried none of the five carbapenemase genes included in our multiplex qPCR panel (blaKPC, blaNDM, blaOXA-48, blaVIM, blaIMP), suggesting alternative resistance mechanisms. Reduced outer membrane permeability—resulting from loss or structural alteration of porins, particularly OmpK35 and OmpK36 in *K. pneumoniae*—is one such mechanism, decreasing antibiotic penetration and intracellular carbapenem concentration and contributing significantly to resistance in Enterobacterales lacking carbapenemase genes (Hamzaoui et al., 2018b; Liang et al., 2024b). Overexpression of multidrug efflux pumps is another: systems such as AcrAB-TolC in Enterobacterales and MexAB-OprM in *P. aeruginosa* actively export antibiotics from the cell, reducing intracellular drug accumulation and potentially acting synergistically with other resistance determinants (Solgi et al., 2021b; Liang et al., 2024b). Carbapenem resistance can also arise from hyperproduction of extended-spectrum β-lactamases (ESBLs) or AmpC β-lactamases combined with porin deficiency, which together can produce clinically significant resistance even without carbapenemase activity (Hamzaoui et al., 2018b)—a mechanism increasingly recognized in Enterobacterales. Finally, some isolates may have carried uncommon carbapenemase genes outside our panel, such as GES, IMI, SME, NMC-A, or other OXA-type variants, which targeted PCR may miss and would require broader approaches, such as whole-genome sequencing, for identification (Liang et al., 2024b). Biofilm formation may also contribute, creating a protective microenvironment that restricts antibiotic penetration, promotes bacterial persistence, and facilitates horizontal gene transfer—particularly relevant in chronic DFUs, where persistent infection and impaired healing favor biofilm-associated antimicrobial tolerance (Li et al., 2025b; Saleem et al., 2025).

Together, these findings highlight the multifactorial nature of carbapenem resistance among GNB in diabetic foot infections. The presence of carbapenem-resistant but carbapenemase-negative isolates underscores the need for broader molecular surveillance, phenotypic characterization, and advanced genomic approaches to fully understand resistance pathways and to guide effective therapy.

The present findings — consistently higher HbA1c among carbapenemase producers across all three time points examined (closest-to-presentation, 12-month mean, and 12-month maximum), a markedly greater proportion of elevated readings (≥8%) in the preceding year, and dose-dependent odds ratios ranging from roughly 8- to 16-fold per 1% increase in HbA1c — situate this cohort within a growing, broadly consistent literature linking chronic glycemic burden to the emergence of multidrug-resistant, and specifically carbapenem-resistant, Gram-negative infection in diabetic foot disease.

Concordance with carbapenemase-specific data. These results closely mirror those of Saleem et al., who examined 200 diabetic foot ulcer (DFU) isolates and found that 51.7% were carbapenemase producers (most commonly *bla*KPC and *bla*NDM), and that patients with carbapenemase-producing MDR infections had poorer clinical outcomes, including higher HbA1c levels (mean 9.1%) and more severe ulcers, a pattern the authors described as consistent with prior reports from Saudi Arabia and the Middle East linking uncontrolled diabetes to MDR infection risk in DFUs (Saleem et al., 2025). The carbapenemase-producing group’s HbA1c range in the present study (8.45–8.81%) falls within this same general envelope, while the non-producer comparator (6.96–7.34%) is meaningfully better controlled — supporting that the observed separation reflects genuine glycemic divergence between subgroups rather than a skewed reference population.

Mechanistic plausibility. The longitudinal pattern reported here — where sustained glycemic burden over the preceding year yields even larger odds ratios than the single value closest to presentation — is well supported by the underlying mechanisms. Darwitz and colleagues’ review of diabetes-associated bacterial infection describes a “triple threat” of altered tissue nutrient availability, immune suppression, and antibiotic treatment failure; hyperglycemia elevates tissue glucose, which many bacterial pathogens preferentially use as a carbon source, and promotes biofilm formation and tissue adherence that contribute to infection chronicity (Darwitz et al., 2024). The same review notes that diabetes-associated immune suppression alters neutrophil chemotaxis, cytokine profiles, and the oxidative burst required for bacterial killing, and that this immune impairment, combined with the polymicrobial diabetic wound microenvironment, may actively promote the emergence of multidrug-resistant strains (Darwitz et al., 2024). This provides biological plausibility for why *cumulative* rather than momentary glycemic exposure would show the strongest association with carbapenemase acquisition.

Consistency with broader MDR risk-factor literature. Several independent DFU cohorts identify HbA1c or glycosylated hemoglobin as a correlate of MDR (not exclusively carbapenemase-producing) infection alongside other clinical variables. Yan et al., in a retrospective study of 180 DFU patients, found that glycosylated hemoglobin, along with BMI, fasting blood glucose, ulcer course and size, peripheral neuropathy, peripheral vascular disease, osteomyelitis, and prior antibiotic use, was significantly associated with MDR bacterial infection, and that leukocyte count and neutrophil ratio were lower in MDR-infected patients, suggesting host immune impairment (Yan et al., 2022). Similarly, Liu et al., in a larger cohort of 348 DFU patients, identified previous hospitalization, ulcer size >4 cm², surgical therapy, and CRP as independent risk factors for MDRO infection on multivariable analysis (Liu et al., 2022). These studies underscore that while your unadjusted odds ratios for HbA1c are substantial, other correlated variables (ulcer duration, prior antibiotic exposure, hospitalization) are plausible confounders or mediators that a multivariable model would need to address.

A unifying immunometabolic framework. Acierno et al.’s recent narrative review frames the relationship between metabolic dysfunction and MDR infection as bidirectional, mediated by chronic low-grade inflammation, insulin resistance, gut microbiota dysbiosis, and antibiotic-driven expansion of the resistome, with MDR infection itself capable of worsening subsequent metabolic control (Acierno et al., 2025). This bidirectionality is a useful caveat for interpretation: while sustained hyperglycemia plausibly drives susceptibility to carbapenemase-producing organisms through impaired immune clearance and a more permissive wound microenvironment, recurrent or severe resistant infection could independently worsen glycemic control through stress hyperglycemia and repeated hospitalization, a loop that unadjusted, single-timepoint-referenced analyses cannot fully disentangle.

Interpretive implications. Taken together, this literature supports treating your findings as a real, biologically coherent association rather than an artifact, while reinforcing the appropriateness of your caution regarding unadjusted observational estimates. The finding that 12-month mean and maximum HbA1c values have larger effect sizes than the single value closest to presentation is particularly persuasive, since these antecedent measures are less susceptible to reverse causation by the acute presenting infection itself. Future analyses adjusting for ulcer duration/size, prior antibiotic exposure, and hospitalization frequency — variables repeatedly identified as MDR risk factors in this literature — would help clarify whether sustained glycemic burden operates as an independent driver of carbapenemase-producing infection or acts primarily through these correlated clinical pathways.

### Strengths and limitations

This study has several strengths. It provides integrated phenotypic and molecular characterization of carbapenemase-producing GNB in DFUs within a tertiary-care setting and links these microbiological findings to key clinical and metabolic parameters. Use of deep tissue specimens—the diagnostic gold standard—enhanced pathogen yield and minimized contamination bias, while combining phenotypic screening with targeted PCR enabled robust genotype–phenotype correlation and improved detection accuracy.

Certain limitations should also be acknowledged. The single-center design and moderate sample size may limit generalizability to other regions or healthcare settings, and the cross-sectional design precludes causal inference regarding the relationship between poor glycemic control and acquisition of carbapenemase-producing organisms. Molecular testing was limited to five carbapenemase genes, so other resistance mechanisms (e.g., porin loss, efflux pumps, or less common carbapenemases) were not directly assessed, and data on prior antibiotic exposure and longitudinal outcomes were similarly limited. Future multi-center prospective studies with expanded molecular profiling and clinical follow-up are warranted to validate and extend these findings.

Taken together, our findings identify poor glycemic control as an important, modifiable risk factor for infection with carbapenemase-producing organisms, whereas Wagner grading alone appears insufficient to predict resistance profiles. Integrated strategies combining metabolic optimization, early microbiological diagnosis, and targeted antimicrobial stewardship are therefore essential to improving outcomes in DFUs, and prospective multicenter studies are warranted to clarify the causal relationship between glycemic status and acquisition of carbapenem-resistant pathogens.

## Conclusion

This study highlights a high burden of multidrug-resistant Gram-negative bacilli in diabetic foot ulcer infections, with carbapenem resistance driven largely by the *bla*KPC and *bla*NDM genes. *Klebsiella pneumoniae* predominated, confirming the shift toward Gram-negative pathogens in advanced infections, and poor glycemic control was significantly associated with carbapenemase-producing isolates, identifying hyperglycemia as an important modifiable risk factor.

The lack of association with Wagner grade suggests that antimicrobial resistance is shaped more by metabolic status and antimicrobial pressure than by ulcer depth. These findings underscore the need for integrated strategies combining optimal glycemic control, early microbiological diagnosis, routine carbapenemase screening, and robust antimicrobial stewardship. Further multi-center prospective studies are warranted.

## Data Availability

The raw data supporting the conclusions of this article will be made available by the authors, without undue reservation.

## Supplementary material

Supplementary Table 1 MIC breakpoints used for antimicrobial resistance interpretation according to CLSI M100 (35th edition, 2025). Breakpoints were interpreted according to Clinical and Laboratory Standards Institute (CLSI) M100, 35th edition (2025). * Colistin breakpoints were interpreted according to CLSI/EUCAST recommendations.

Supplementary Table 2 Association between specimen type and phenotypic carbapenemase production. Chi-square test = 0.10. P value = 0.992.
