# Correction: Molecular epidemiology of carbapenemase-producing gram-negative bacilli in diabetic foot infections: association with glycemic control

**DOI:** 10.3389/fcimb.2026.2030828

**Published:** 2026-10-29

**Authors:** Gamal Othman, Abdelnaser A. Badawy, Rasha Hamed Al-Serwi, Maysaa El Sayed Zaki, Noha Tharwat Abou El-khier, Nashwa M. Alkassaby, Mohamed Saleh Ismail

**Affiliations:** 1 Department of Basic Medical Sciences, College of Medicine, AlMaarefa University, Riyadh, Saudi Arabia; 2 Department of Biochemistry, Faculty of Medicine, Northern Border University, Arar, Saudi Arabia; 3 Department of Basic Dental Sciences, College of Dentistry, Princess Nourah bint Abdulrahman University, Riyadh, Saudi Arabia; 4 Clinical Pathology-Mansoura Faculty of Medicine, Mansoura University, Mansoura, Egypt; 5 Medical Microbiology and Immunology- Mansoura Faculty of Medicine, Mansoura University, Mansoura, Egypt

**Keywords:** antimicrobial stewardship, blaKPC, BlaNDM, carbapenem resistance, diabetic foot ulcer, gram-negative bacilli, HbA1c, *Klebsiella pneumoniae*

An incorrect number was provided for the “Deanship of Scientific Research at Northern Border University, Arar, Saudi Arabia”. The correct number is “NBU-FFR-2026-52-03”.

The original version of this article has been updated.

