# Supplementary material for: Molecular epidemiology of carbapenemase-producing gram-negative bacilli in diabetic foot infections: association with glycemic control
Source: Front Cell Infect Microbiol. 2026 Oct 5;16:1847743. doi: 10.3389/fcimb.2026.1847743 (PMC13677365; doi:10.3389/fcimb.2026.1847743)
Supplement: Supplementary file 1 [file Table1.docx]

# Supplementary Table S1: MIC breakpoints used for antimicrobial resistance interpretation according to CLSI M100 (35th edition, 2025).

| Antibiotic | Susceptible (µg/mL) | Resistant (µg/mL) |
| --- | --- | --- |
| Imipenem | ≤1 | ≥4 |
| Meropenem | ≤1 | ≥4 |
| Ceftazidime | ≤4 | ≥16 |
| Cefepime | ≤2 | ≥16 |
| Piperacillin–Tazobactam | ≤16/4 | ≥128/4 |
| Ciprofloxacin | ≤0.25 | ≥1 |
| Levofloxacin | ≤0.5 | ≥2 |
| Amikacin | ≤16 | ≥64 |
| Colistin* | ≤2 | >2 |

Footnotes:

Breakpoints were interpreted according to Clinical and Laboratory Standards Institute (CLSI) M100, 35th edition (2025).

* Colistin breakpoints were interpreted according to CLSI/EUCAST recommendations.
