# Supplementary material for: Molecular epidemiology of carbapenemase-producing gram-negative bacilli in diabetic foot infections: association with glycemic control
Source: Front Cell Infect Microbiol. 2026 Oct 5;16:1847743. doi: 10.3389/fcimb.2026.1847743 (PMC13677365; doi:10.3389/fcimb.2026.1847743)
Supplement: Supplementary file 2 [file Table2.docx]

# Supplementary Table S2: Association between specimen type and phenotypic carbapenemase production

| Sample type | Total (n) | Carbapenemase producer n (%) | Non-producer n (%) |
| --- | --- | --- | --- |
| Tissue | 61 | 52 (85.2%) | 9 (14.8%) |
| Swab | 46 | 38 (82.6%) | 8 (17.4%) |
| Pus | 30 | 25 (83.3%) | 5 (16.7%) |
| Bone biopsy | 13 | 11 (84.6%) | 2 (15.4%) |
| Total | 150 | 126 (84.0%) | 24 (16.0%) |

Chi-square test = 0.10

P value = 0.992
